# Penny for your thoughts: A review and proposed model of socioeconomic status, working memory and worry

**DOI:** 10.1111/bjdp.70056

**Published:** 2026-06-07

**Authors:** Colton L. Hunter

**Affiliations:** ^1^ Department of Psychological Science University of Arkansas at Little Rock Little Rock Arkansas USA

**Keywords:** cognition, income, socioeconomic status, working memory, worry

## Abstract

Socioeconomic status is a complex psychological construct linked to a number of professional and personal barriers, including cognitive deficits. Current explanations of the link between socioeconomic status and cognition focus primarily on influences of external factors on development, with less attention paid to internal psychological mechanisms operating in the present. In this review, I propose The Bucket Model of Socioeconomic Working Memory Disparities as a complementary framework, which attributes a portion of socioeconomic deficits in working memory to increased worry and social comparison. To accomplish this, I first summarize research linking socioeconomic status to cognitive outcomes. I then explore the role of working memory specifically in these links. Finally, I define The Bucket Model of Socioeconomic Working Memory Disparities, exploring possible pathways by which worry and social comparison divert resources away from working memory processes.

**Key points**

- Socioeconomic status is linked to a number of barriers to success via cognition.
- Working memory in particular is important and impacted by socioeconomic status.
- A novel model, The Bucket Model of Socioeconomic Working Memory Disparities, is proposed.
- This model attributes a portion of socioeconomic deficits in working memory to increased worry, which passively occupies working memory resources and shifts attention.

In western society, money shapes nearly every aspect of human existence. It at least in part determines the resources, hardships and opportunities present throughout life; may influence dynamics within social interactions; is associated with differences to the biological systems within the human body; and is linked to both the mechanisms and overall functions of human cognition (Chevalier et al., 2013; Duncan & Magnuson, 2012; Hackman et al., 2014; C.‐Y. S. Lee et al., 2009). Consequently, socioeconomic status is a complex psychological construct that seeks to encompass the differences in resources, opportunities and social status seen throughout society based on an individual's income, education and occupational prestige. Measures of socioeconomic status also typically reflect an individual's subjective views of their status relative to both their peers and within society as a whole. This subjective comparison, along with the number of increased stressors and barriers to success associated with socioeconomic inequality, can create a near‐constant worry for socioeconomically disadvantaged individuals (Schmitt et al., 2023; Sheehy‐Skeffington, 2020). However, whether this worry might directly explain some of the other potential effects of socioeconomic status (e.g., on executive functions) has not been fully explored.

Executive functions, which include working memory, inhibition and cognitive flexibility, are a set of higher order processes that govern goal‐directed thoughts and behaviours, and they play a critical role in creating or maintaining physical and mental health, professional success and overall quality of life (A. Diamond, 2013; Miyake & Friedman, 2012). Working memory in particular is necessary for planning, reasoning and reading comprehension (Alloway, 2006; Alloway et al., 2004; Owen et al., 1996), as doing so requires the ability to hold mental representations of items and draw connections between them. These skills form the backbone of critical thought and are crucial to human functioning in an increasingly complex world. Yet working memory deficits have been consistently observed among low socioeconomic status individuals (Duncan & Magnuson, 2012; Hackman et al., 2014, 2015; Hunter & Shields, 2022; Sheehy‐Skeffington, 2020; L. Zhang et al., 2019). Current explanations for this association have largely focused on developmental differences, with environmental adversity predicting differences in neural development, ultimately contributing to disparities in working memory across the lifespan. However, while developmental pathways are well established, they may not fully account for the role of internal psychological factors such as worry and social comparison. The present review argues that these concurrent factors constitute an additional pathway to working memory deficits, one that can operate independently of, and alongside, developmental influences.

The purpose of the current review is to examine the interactions between measures of socioeconomic status, finance‐related worry and working memory. In doing so, I hope to propose an integrative model of working memory disparities across socioeconomic classes due to socioeconomic worry and rumination. To achieve this goal, I first provide an overview of working memory components and processes. Second, I summarize contemporary research examining socioeconomic influences on cognition. Third, I propose an integrative model of socioeconomic status, worry and working memory, which I call the *Bucket Model of Socioeconomic Working Memory Disparities*. This model proposes that socioeconomic status‐related deficits in working memory can be attributed to socioeconomic worries diverting working memory resources away from task‐related stimuli. Finally, I evaluate existing explanations for the association between socioeconomic status and working memory performance in comparison to the proposed model.

## THE ROLE OF WORKING MEMORY

Working memory is the ability to hold information in mind and integrate it with old information when appropriate (Shields et al., 2015, 2021). It has been studied extensively in connection to a number of skills, including problem solving, reading comprehension, information processing and academic learning (Alloway, 2006; Alloway et al., 2004; Bechara et al., 1998; Johann et al., 2020; Owen et al., 1996; Radüntz, 2020; Rakow et al., 2010; Seigneuric et al., 2000). However, definitions of working memory have evolved over time, implicating subprocesses and parameters that govern the means and effectiveness of one's working memory. In the current section, I examine the specific components of working memory to explore the distinct influences of socioeconomic status on these components.

### Working memory components and processes

Representations, or items, in WM may vary in quantity (capacity) and quality (precision) (Luck & Vogel, 2013; Ma et al., 2014). Consequently, forgetting may be a degradation of quantity (rapid decay of the representation) or quality (slow decay of the representation over time). Differences in the limitations of working memory capacity between individuals reflect differences in cognitive complexity and are a marker of socioeconomic status‐related deficits (Hackman et al., 2014; L. Zhang et al., 2019). Broadly, two models of visual working memory have emerged: fixed capacity models and continuous resource‐allocation models. Fixed capacity models propose that working memory has a limited number of available slots, which store a number of discrete representations with fixed precision (Cowan, 2001; Luck & Vogel, 1997; Vogel et al., 2001). In contrast, resource‐allocation models describe working memory in terms of a continuous pool of cognitive resources. Additionally, resource allocation based models propose that working memory precision is a product of working memory capacity (Bays et al., 2009, 2011; Bays & Husain, 2008; Ye et al., 2017; Yoo et al., 2018). The precision with which an item is stored is determined by the proportion of total cognitive resources allotted to it: The more items held in capacity at one time, the more cognitive resources are divided between them, and the worse precision is. Because individuals completing working memory tasks do not generally know which item will be probed, resource‐allocation models presume that resources are equally allocated between items, resulting in the precision deficit as set size increases. Additionally, resource‐allocation models attribute differences in working memory performance to differences in maximum working memory resources. However, although resource‐allocation models explain the experimental data well, they assume a fixed resource budget for each item (van den Berg & Ma, 2018). Working memory resources in daily life are not always allocated equally across all bits of information (Yoo et al., 2018). One possible model to address this deficit views working memory capacity and precision through the lens of expected performance compared with cost of encoding (van den Berg & Ma, 2018). At encoding, there is a trade‐off between the high cost of encoding and the behavioural benefits of maintaining the information with high precision. Decreases in precision as set size increases are then thought to be the result of an individual attempting to make this trade‐off optimally.

## SOCIOECONOMIC STATUS AND EXECUTIVE FUNCTION

When considered as a whole, there is clear evidence that socioeconomic inequality creates barriers to success and well‐being across a number of domains. Although investigating each of these pathways is critical, executive function is especially important in predicting overall achievement. Executive function plays a critical role in professional development and success (A. Diamond, 2013; Gioia et al., 2010; Hunter & Shields, 2022). Consequently, deficits in executive function have been associated with a wide variety of socioeconomic factors and pathways. In the current section, I examine connections between socioeconomic factors and executive function as a whole within the current literature.

Links between socioeconomic status and executive function have been studied extensively in childhood. Generally, higher indicators of socioeconomic status (i.e., parental income, education, neighbourhood disadvantage, etc.) are associated with better executive function in children and adolescents (Hackman et al., 2015; Howard et al., 2020; Hunter & Shields, 2022; Last et al., 2018; Lawson et al., 2018; Rosen et al., 2020; Sarsour et al., 2011; Waters et al., 2021). More specifically, critical aspects of a child's home life, such as enrichment opportunities, cognitive stimulation and parental nurturing have been found to aid the development of critical executive functioning (Hackman et al., 2015; Rosen et al., 2020; Sarsour et al., 2011). Low‐income homes typically have fewer opportunities for enrichment along with increased environmental threats, illustrating an intuitive mediator in the relationship between socioeconomic status and executive function in early childhood.

These links between SES and executive function persist long past childhood, though. Socioeconomic status in both childhood and adolescence has been found to influence executive function well into adulthood (Hunter & Shields, 2022; Last et al., 2018; Rosen et al., 2020). These findings therefore suggest that socioeconomic inequality contributes to cognitive barriers to success early in life, and that these impacts are maintained even in the presence of further environmental influences later in life.

However, socioeconomic factors in adulthood influence cognition through their own unique pathways. Within older adults, both higher childhood and adulthood socioeconomic status have been found to uniquely predict better executive function late in life (Jefferson et al., 2011; Lyu & Burr, 2016; Wörn et al., 2017; M. Zhang et al., 2015). When adjusting for education, which is heavily determined by childhood socioeconomic status, current socioeconomic status in older adults is positively correlated with cognitive functioning (Jefferson et al., 2011). Similarly, research examining individual and neighbourhood‐level socioeconomic factors in older adults has found that individual‐level factors more heavily influence cognition (Wörn et al., 2017). Taken together, these findings suggest that childhood and adulthood socioeconomic factors exert cognitive influences through distinct but related pathways.

Evidence for the impact of current socioeconomic status has also been found in middle‐aged and young adults. Executive function in middle‐adulthood has been found to be directly correlated with adult socioeconomic status and indirectly related to childhood socioeconomic status through a combination of education and contributions to adult socioeconomic status (Singhmanoux et al., 2005; Zarantonello et al., 2020). Interestingly, socioeconomic inequality in adulthood may not always predict an *impairment* in executive function. Personal relative deprivation, defined as the belief that you are worse off compared with the people around you, has been associated with greater working memory for non‐emotional social information and worse working memory for non‐social information (L. Zhang et al., 2019). Given that standard working memory tasks typically involve non‐emotional non‐social stimuli (i.e., numbers and letters), it may be the case that socioeconomic deprivation predicts a shift in priority towards cognitive processes related to resource maintenance and social mobility. Further support comes from work examining decision‐making processes. Low socioeconomic status individuals face increased environmental instability and resource scarcity, potentially triggering a shift in cognitive resource allocation towards present goals as opposed to future goals in order to prioritize short‐term financial concerns (Sheehy‐Skeffington, 2020). If this shift towards present goals is indeed due to the influences of socioeconomic worry, previously observed *deficits in working memory may in fact be due to a shift in resource allocation* (or a reluctance to do so) rather than directly reduced working memory capabilities.

### Current mechanisms linking socioeconomic status and working memory

So far, I have defined working memory and its components. I have also reviewed links between socioeconomic status and executive function impairments. Next, I will explore the relation between socioeconomic status and working memory in detail, summarizing existing literature. In doing so, I will examine established mechanisms linking socioeconomic status and working memory processes.

As previously discussed, associations between socioeconomic factors and working memory throughout the lifespan have been extensively studied (Duncan et al., 2017; Duncan & Magnuson, 2012; Evans & Schamberg, 2009; Finn et al., 2017; Gathercole et al., 2004; Hackman et al. 2014, 2015; Howard et al., 2020; Hunter & Shields, 2022; Last et al., 2018; Lawson et al., 2018; K. Lee, 2023; Leonard et al., 2015; Mance et al., 2019; Piccolo et al., 2016; Rosen et al., 2020; Sarsour et al., 2011; Sheehy‐Skeffington, 2020; Sutin et al., 2022; Ursache et al., 2015; Waters et al., 2021; Zarantonello et al., 2020; L. Zhang et al., 2019, 2021). Importantly, the association between socioeconomic status and working memory may also explain many of the associations between socioeconomic status and important life outcomes.

As an example of what was alluded to above, within young children, Waters et al. (2021) found a significant link between both parental education and income‐to‐needs ratio and working memory in children. Furthermore, working memory significantly mediated the link between socioeconomic factors and academic achievement, suggesting not only a basic cognitive impact of socioeconomic factors but also a specific academic disparity. Notably, once prior achievement was included in their model, socioeconomic factors no longer predicted academic success. This is in line with other work that has found no difference in achievement rate across socioeconomic classes in early childhood (Caro et al., 2009; V. E. Lee & Burkam, 2002). Work by Hackman et al. (2014, 2015) has found similar results: Parent education but not neighbourhood disadvantage is associated with poorer working memory, and this association is consistent across late childhood and adolescence.

Importantly, the above findings suggest that socioeconomic disparities in working memory are stable throughout development, rather than being an accumulating effect or a developmental delay. Research examining the developmental impact of deprivation may argue that individuals who experience a lack of stimulation during critical periods of development experience a lower developmental ceiling as opposed to merely a delay (McLaughlin et al., 2014, 2019; Sheridan & McLaughlin, 2014). Although reductions in developmental ceilings have been found in animal models, the specific nature of the effects of deprivation in human subjects is more unclear. Furthermore, if working memory disparities are due to cumulative effects of chronic stress and environmental threats, one would expect these disparities to increase over time; however, this was not the result observed by Hackman et al. (2014), Hackman et al. (2015). This instead suggests that the impact of childhood socioeconomic status on working memory throughout life may primarily be due to consistent social influences within the home. Further evidence for this view comes from prior work conducted by the author. When examining associations between adolescent family income and working memory in adulthood, as well as potential mediators of this association, individual educational attainment as well as parental educational attitudes during adolescence both significantly mediated the longitudinal association between income and working memory (Hunter & Shields, 2022). Additionally, further evidence for the role of cognitive enrichment comes from work examining attention. Infant attention to audiovisual social and non‐social events has been found to mediate the association between socioeconomic status and visual working memory in childhood (Edgar et al., 2023). Furthermore, children from low socioeconomic families display poorer sustained attention as well as disrupted neural responses to attentional stimuli (Hampton Wray et al., 2017). Given the well‐established overlap between attention and working memory (A. Diamond, 2013; van Ede & Nobre, 2023), this highlights a pathway through which socioeconomic environment may impair visual attention, which may then in turn result in poorer working memory. Taken together, these findings suggest that socioeconomic disparities in working memory are shaped by multiple converging pathways: developmental influences on neural structure and function, as well as ongoing social and environmental factors such as family dynamics, parental attitudes and the quality of cognitive enrichment throughout childhood.

In contrast to work examining childhood socioeconomic status, which primarily focuses on the roles of enrichment and deprivation, work examining the influence of adult socioeconomic status on working memory typically focuses on the role of financial stressors as well as social comparison. Within undergraduate students, both financial concerns and social belonging have been linked to academic success, with working memory in particular mediating these links (Reid et al., 2020). Notably, this research accounted for background variables such as parental socioeconomic status and prior academic achievement, revealing an important influence of concurrent socioeconomic factors. Separately, a review of nine studies examining socioeconomic status, stress and executive function conducted by Piccolo et al. (2014) found a consistent influence of both childhood and adult socioeconomic factors. Stress acted as a mediator for relationships between both and working memory in adulthood. The role of stress as a mediator then suggests the possibility of two distinct pathways by which factors in early life and recent life factors separately lead to greater stress, which in turn reduces working memory (Olver et al., 2015; Porcelli et al., 2008; Schoofs et al., 2008, 2013; Shields et al., 2016, 2017, 2019; Zandara et al., 2016). Although it is likely that stress plays a significant role, there is also clear evidence of subjective influences. A pair of studies conducted by Zhang et al. examined working memory as a function of relative deprivation, the belief that you are worse off compared with the people around you (L. Zhang et al., 2019, 2021). Individuals with greater subjective feelings of deprivation displayed better working memory for neutral social stimuli at the cost of working memory for non‐social stimuli. Additionally, induced feelings of relative deprivation were found to result in reduced ability to ignore distracting threat‐related faces in a visual working memory task. Importantly, this work illustrates that subjective socioeconomic factors are more specifically linked to a shift in attention and working memory, rather than an overall reduction in capabilities. This finding also contributes to some of the difficulty in discerning the effects of socioeconomic status in adulthood on working memory, as there is clear evidence for the role of developmental factors in childhood as well as evidence for the role of stress and social comparison.

## THE BUCKET MODEL OF SOCIOECONOMIC WORKING MEMORY DISPARITIES: SOCIOECONOMIC WORRY AND RESOURCE ALLOCATION

Existing models of socioeconomic differences in cognition focus almost exclusively on developmental influences. Current models also primarily focus on objective socioeconomic outcomes and their environmental impact; however, as previously discussed, internal concerns and social dynamics can be equally as important links between socioeconomic status and cognition (Aspinwall & Taylor, 1993; Huang et al., 2016; Reid et al., 2020; Rios & Zautra, 2011; Wang & Liu, 2021; L. Zhang et al., 2019, 2021; M. Zhang et al., 2015). Notably, there is a substantial body of clinical work examining associations between rumination, executive function and socioeconomic status.

Rumination, defined as persistent, repetitive dwelling on negative thoughts without resolution (Olatunji et al., 2013), is a hallmark of both depressive and anxiety disorders. Indeed, rumination has been explored as a transdiagnostic factor, given its association with the development of both categories of psychological disorder (McLaughlin & Nolen‐Hoeksema, 2011; Olatunji et al., 2013). These associations have led for much work examining the role of rumination in explaining links between anxiety and depression and deficits in executive functions. However, the nature of these links is not clear cut. At a meta‐analytic level, negative associations have been found between rumination and specific executive functions, including set‐shifting and inhibition (Vălenaș & Szentágotai‐Tătar, 2017; Yang et al., 2017). Notably, within this work and other studies, rumination was not found to be significantly associated with working memory (Connolly et al., 2014; Vălenaș & Szentágotai‐Tătar, 2017; Yang et al., 2017). However, as previously discussed working memory is not monolithic, and rumination may differentially influence specific working memory processes. For example, individuals high in ruminative tendencies experience greater difficulty in removing outdated items from working memory, suggesting a deficit in working memory gating (Bruning et al., 2023). Similarly, aspects of rumination have been found to be positively correlated with performance on an antisaccade task, but negatively correlated with 2‐back performance (Nishimura et al., 2022). Nevertheless, links between rumination and executive function have led to work explaining socioeconomic status‐related deficits in cognition via rumination. Lower socioeconomic status is associated with an increased risk of anxiety and depression, as well as increased levels of rumination (de Bruijn & Antonides, 2020; Szabo et al., 2022; Talavera et al., 2018; Wu et al., 2022). Within children and adolescents, links between executive function, rumination and socioeconomic status have been observed such that greater levels of rumination are predictive of poorer executive function and greater risk of developing psychopathology longitudinally (McNeilly et al., 2021; Sutin et al., 2022). In work examining college students, anxiety and depression symptoms, including rumination, were predictive of poorer set‐shifting and inhibitory control (Gómez et al., 2023). It is worth noting that this work has examined executive function overall, with little work devoted to examining associations between socioeconomic status, rumination and working memory.

Within financially disadvantaged individuals, worry may influence working memory more than rumination. Worry and rumination are distinct psychological concepts, with worry referring to a chain of negative thoughts regarding an event whose outcome is uncertain but potentially adverse, and rumination referring to behaviours and thoughts that passively reflect on one's depressive symptoms (Becker et al., 2003; Borkovec et al., 1983; Hoyer & Gloster, 2009; Nolen‐Hoeksema, 1991). Consequently, the two also differ in predicting psychopathology, with worry being a stronger predicter of both anxiety and depression than rumination alone (Becker et al., 2003; Hoyer & Gloster, 2009). These differences also exist cognitively. State‐based active worrying has been found to reduce working memory capacity, gating of new information in working memory, and overall working memory performance (Crowe et al., 2007; Hayes et al., 2008; Sari et al., 2017; Stout et al., 2015). Additionally, experimentally reduced worry has been found to significantly reduce working memory capacity (Curci et al., 2013; Smith, 2013), suggesting a causal relationship between worry and working memory resources. Socioeconomic status is negatively correlated with worry, such that those of lower socioeconomic status consistently experience greater levels of worry across a variety of domains. Income, past financial changes and ability to make ends meet have been found to be negatively correlated with worry, such that lower socioeconomic status is predictive of greater worry (de Bruijn & Antonides, 2020). Similar evidence has been found within socioeconomically disadvantaged college students, with subjective social status also implicated as a significant predictor (Wu et al., 2022). Evidence also exists for greater health‐care‐related worry for low socioeconomic individuals (Zhou et al., 2025), greater academic worry for socioeconomically disadvantaged children (Gibbs & Kenealey, 2022), and a significant link between childhood socioeconomic status and general worry in young adults (Shair et al., 2025). Moreover, greater levels of worry have been implicated as a causal pathway in explaining links between socioeconomic status and adverse mental health outcomes (Knifton & Inglis, 2020; Weissman et al., 2020). This may therefore suggest a pathway by which greater levels of worry contribute to reduced working memory capacity due to the increased number of financial stressors that low socioeconomic status individuals experience. However, little work has examined this directly. Work has examined interactions between socioeconomic status and worry within an academic setting, finding a significant interaction between the two on academic performance with an adolescent sample (Gibbs & Kenealey, 2022). Additionally, work has examined the effects of upward social comparison, a common social stressor within disadvantaged individuals, finding that it impairs working memory capacity (Suran et al., 2021).

Work examining the influence of worry may provide a novel pathway to examine links between socioeconomic status and working memory via socioeconomic worry. I therefore propose *the Bucket Model of Socioeconomic Working Memory Disparities*, an integrative model of the role of past and current socioeconomic status on working memory. Suppose we view working memory as a bucket, which we can fill with a number of items that are held in working memory (Figure 1).

**FIGURE 1:**
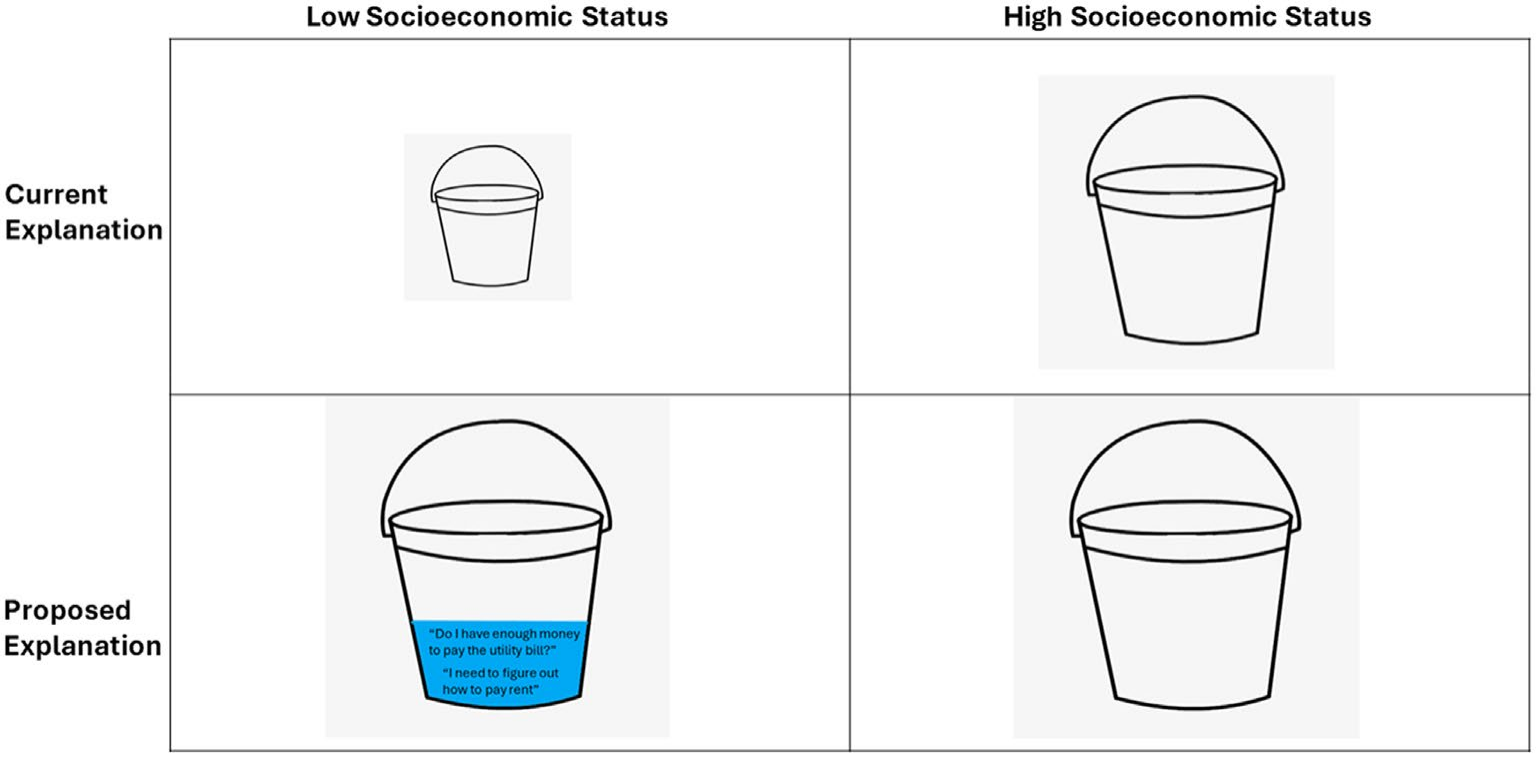
Proposed “bucket” analogy of working memory deficits. Current explanations propose that low‐SES individuals have reduced working memory capacity due to reduced prefrontal cortex function (“a smaller bucket”). The proposed explanation is that they have reduced capacity due to pre‐allocated resources (“a half‐full bucket”).

Previous models would suggest that low socioeconomic status individuals have a smaller bucket (i.e., fewer available cognitive resources due to an impaired prefrontal cortex, resulting in reduced working memory capacity). I instead propose that individuals across socioeconomic classes have similarly sized “buckets”, but that lower socioeconomic status individuals simply have a bucket that is “half‐full” (i.e., cognitive resources are diverted towards worry, rumination and potential threats). Resource rational models of working memory propose a trade‐off between the cost of expending cognitive resources to encode information and the behavioural benefits of doing so. For low‐income individuals, there is a high behavioural benefit to retaining information related to socioeconomic worry (e.g., remembering to pay bills, avoiding social threats, ensuring access to food and shelter) relative to the neutral information typically used in working memory tasks. This could potentially result in socioeconomic status exerting influences on the importance of neural cost relative to behavioural cost, influencing the value of the weight (*λ*) parameter within resource rational models. Consequently, the common finding throughout the literature of low socioeconomic status individuals having poorer working memory capacity and precision could instead be attributed to a refusal to divert cognitive resources from socioeconomic worries to task stimuli rather than simply fewer cognitive resources.

### Evidence for the Bucket Model of Socioeconomic Working Memory Disparities

The idea that socioeconomic status impairs working memory via socioeconomic worry has substantial support within the surrounding literature. The notion that preoccupation in working memory from stressors and other external factors may limit performance has been found by a number of authors (Baddeley, 2013; Hou & Cai, 2022; Levey et al., 1991; Luethi, 2008; Yuan et al., 2016). Disadvantaged individuals face unique stressors and pressures that are constantly in their minds. For example, financial precarity and material insecurity have been found to predict a shift towards present‐focused thinking (Schmitt et al., 2023; Sheehy‐Skeffington, 2020), suggesting that lower SES individuals consider future problems more heavily in the present moment and potentially devote more cognitive resources towards them out of necessity. This would result in subsequent task‐related items being deemed less relevant and less cost‐efficient to spend resources on. Additional support comes from research examining the effects of worry on working memory parameters. As previously discussed, low socioeconomic status individuals experience increased worry from environmental stressors and social comparison. Worry in turn can significantly impair working memory. Worry uses up cognitive resources, diverting them towards thoughts and concerns about potential problems and away from task‐relevant processes (Hayes et al., 2008; Leigh & Hirsch, 2011; Sari et al., 2017). This in turn significantly reduces working memory capacity and task attention, with greater levels of worrying associated with more severe impairments. These influences are not only seen in adults. Within children, internalizing behaviours such as worry and rumination are predictive of poorer executive function through similar mechanisms (K. Lee, 2023; Sutin et al., 2022; Wang & Liu, 2021). These findings therefore provide a pathway through which socioeconomic status in both children and adults leads to greater worry about stressors, which in turn impairs working memory by diverting cognitive resources towards rumination.

Similar pathways can be observed in research examining the effects of stress on working memory. Acute stress exposure prior to a working memory task has been found to impair working memory performance (Olver et al., 2015; Porcelli et al., 2008; Schoofs et al., 2009; Shields et al., 2016; Zandara et al., 2016). This effect is generally attributed to a shift in resource allocation towards stressor‐relevant processes, as evolutionarily these processes are most necessary for survival. Consistent with this explanation, acute stress has been found to impair task‐related attention and cognitive flexibility (Plessow et al., 2011; Shields et al., 2016). Similarly, recent life stress (i.e., stressful events experienced within the past two weeks) has been found to be predictive of lower working memory capacity but not precision, potentially through narrowing attentional focus (Shields et al., 2019). This finding remained consistent even when accounting for lifetime stress exposure, suggesting a unique pathway regarding more contemporary stressors as opposed to merely chronic effects. Additionally, even recalling a prior stressful experience can shift attention, as writing about a prior unresolved stressful experience has been found to impair overall working memory performance as well as attention to the task (Hunter & Shields, 2024). Taken together, these findings suggest the proximal mechanism by which worry and stress impair working memory is attentional, with worry recruiting selective attention towards threat‐relevant content. Because attention and working memory share common cognitive resources, this leaves fewer resources available for encoding and maintaining task‐relevant information. Inhibitory control then plays a moderating role, with individuals better able to suppress intrusive worry‐related thoughts potentially showing smaller working memory impairments.

Notably, prior work has proposed similar mechanisms in regards to broader cognitive functions (Mani et al., 2013). Experimentally induced socioeconomic worry, done so via low vs. high‐cost financial decisions, preferentially reduced cognitive performance in poorer participants but not richer participants. Mani et al. attributed these findings to a cognitive load imposed by poverty, which reduces attention and effort. However, although cognitive load may explain the impact of socioeconomic worry on broader cognition, it might not explain influences on working memory processes specifically. Recent work has examined the role of activity‐silent synapses in working memory. These models suggest that access to information within working memory can be maintained in the absence of typical ongoing neural activity through lingering patterns of synaptic connectivity (Barbosa et al., 2020; Oberauer & Awh, 2022). It is through these mechanisms that socioeconomic worries may remain within working memory without being directly attended to, influencing working memory processes without necessarily sapping broader attentional resources. The maintenance of socioeconomic worries via activity‐silent working memory might also better explain other lab‐based cognitive findings, as Mani et al.'s proposed mechanism of attentional capture and cognitive load would suggest a constant, intentional focus on socioeconomic worries as opposed to a latent background process. This constant focus would also predict global deficits in cognition from worry. However, work examining both state and trait level worry has found certain cognitive functions are positively associated with worry. Specifically, threat of shock has been found to be predictive of improved cognition at multiple levels (Newman & Llera, 2011; Robinson et al., 2013). Additionally, induced worry has been found to enhance theory‐of‐mind reasoning for negatively valanced stimuli (Zainal & Newman, 2018). A worry‐induced shift in resources, and the potential cognitive enhancement it might bring, are also consistent with strengths‐based approaches to studying disadvantaged communities, which examine adversity and resilience through a lens of strengths and benefits rather than purely deficits (Alper et al., 2025; Silverman et al., 2023). Attentional shifting and silent‐activity synapses are not mutually exclusive and may describe distinct stages of the same process, with active worrying initially recruiting attentional resources towards financial concerns. Over time, these concerns may become encoded via activity‐silent synaptic mechanisms, allowing them to persist in working memory via latent background processes. This would potentially result in financial worry not producing global cognitive deficits while still selectively interfering with working memory task performance by occupying working memory specific resources.

In summary, low socioeconomic status is associated with a shift towards present‐focused thinking, resulting in greater cognitive resources and value being allocated towards financial problems and stressors and increasing the frequency of finance‐related worry. Through active attentional capture, worry initially recruits cognitive resources towards financial concerns and away from task‐relevant processes, resulting in observed working memory impairments. Over time, these concerns may become encoded via activity‐silent synaptic mechanisms. Together, both mechanisms suggest that financial worry influences working memory selectively, and that this inhibition operates through both active attentional and passive synaptic mechanisms.

### Alternate explanations for working memory deficits

Socioeconomic‐related deficits in working memory are primarily explained through developmental pathways. Parental socioeconomic factors such as income and education afford their children a certain quality of life, leading to increased enrichment opportunities, reduced risk of deprivation and fewer environmental stressors. Broadly, lower socioeconomic status has been consistently linked to reduced activity and growth in the prefrontal cortex, the brain region primarily implemented in working memory processes (McLaughlin et al., 2014, 2019; Sheridan et al., 2022; Sheridan & McLaughlin, 2014). More specifically, environmental factors associated with poorer socioeconomic status, such as more prevalent threats and increased chance of deprivation, have been examined as predictors of neural development. Through this integrated view, a model exists within the current literature by which socioeconomic status predicts the prevalence of threats and deprivation in the environment, called the Dimensional Model of Adversity (McLaughlin et al., 2014) (Figure 2). Each in turn exerts unique influences on neural development, which lead to changes in working memory and other executive functions.

**FIGURE 2:**
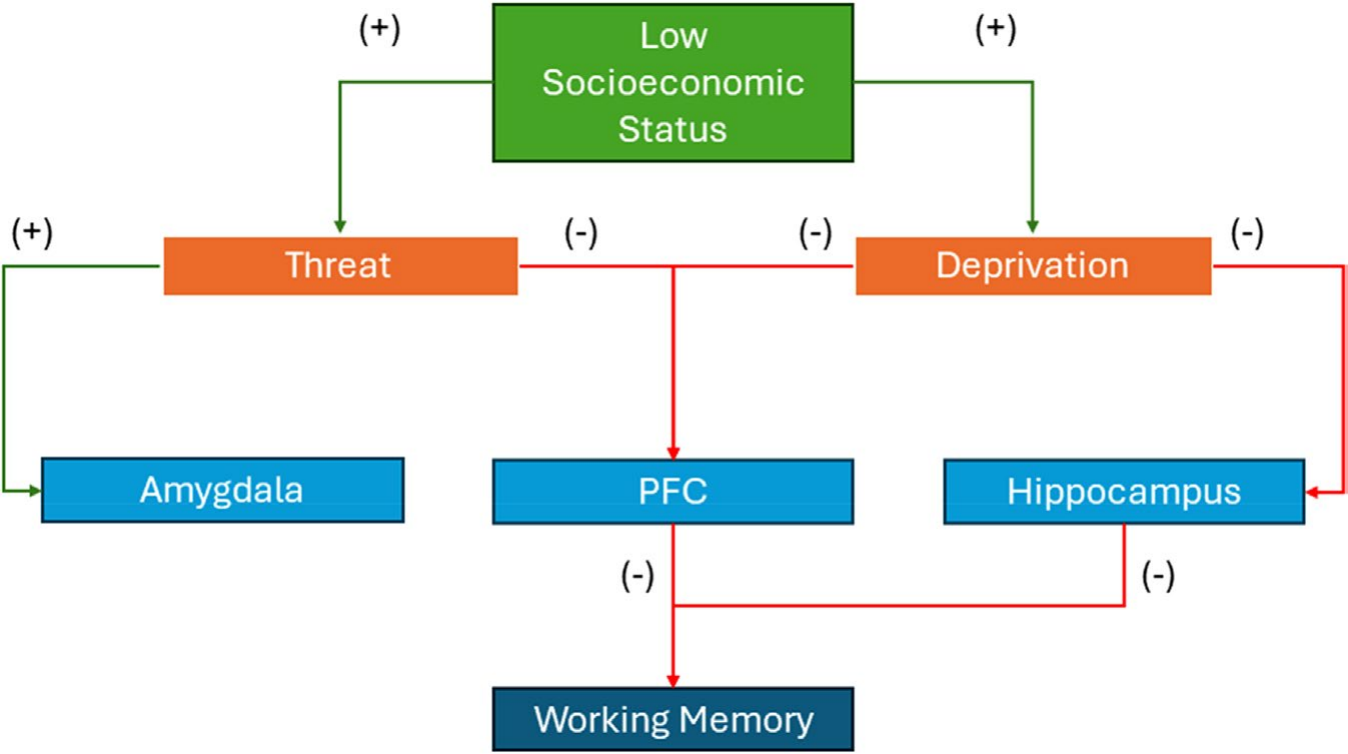
The dimensional model of adversity. Childhood adversity can be categorized along dimensions of threat and deprivation, with each exerting unique influences on neural development.

McLaughlin's model seeks to examine socioeconomic disparities among dimensions of threat (the presence of detrimental environmental factors) and deprivation (the absence of beneficial environmental factors). Both deprivation and threat exert unique influences on the prefrontal cortex in development. For example, research within non‐human animals found that sensory deprivation significantly increased synaptic pruning, the removal of over‐produced connections in the brain (Bennet et al., 1974; Wiesel & Hubel, 1965). Importantly, synaptic pruning has been implicated as a key mechanism by which experiences shape neural structures and, by extension, functions (Changeux & Danchin, 1976). Additionally, global deprivation within rats has been associated with reduced dendritic branching and cortical thickness; however, these effects were later reversed by the addition of environmental enrichment (M. C. Diamond et al., 1972). Within humans, similar results can be seen regarding a lack of cognitive inputs rather than merely sensory. Psychosocial deprivation in early childhood is associated with greater cortical thinning in the lateral and medial prefrontal cortices. Similarly, children who are neglected or who are raised in foster care display a similar pattern of changes: reduced thickness of the prefrontal cortex and disruptions to prefrontal cortex function (McLaughlin et al., 2014, 2019; Sheridan et al., 2022). These changes in neural structure and function are predictive of impaired cognition globally (Dickerson et al., 2008; Tuladhar et al., 2015). Taken together, these findings suggest that environmental deprivation predicts impaired working memory via influences on synaptic pruning and one's ability to form vital social and cognitive associations throughout development.

However, low socioeconomic status is generally predictive of greater risk of threat as well as deprivation (Baum et al., 1999; Evans & Kantrowitz, 2002; Hunter & Shields, 2022; Kim & Park, 2015; Mance et al., 2019). Within rodents, early life threat exposure is predictive of reduced dendritic branching and impaired hippocampus function in adulthood, along with reduced cortical thickness in the ventromedial prefrontal cortex (Eiland et al., 2012; Ivy et al., 2010; Sarro et al., 2014; Toledo‐Rodriguez & Sandi, 2007). Additionally, early threat has been found to predict greater baseline amygdala activity, suggesting an elevated sensitivity to threat along with reduced cognitive capabilities (Raineki et al., 2012). Extending these findings to humans, children exposed to violence and abuse in early childhood exhibit similar changes: reduced hippocampal function in adulthood, reduced ventromedial prefrontal cortex volume and enhanced sensitivity to threats (Hanson et al., 2010; McCrory et al., 2011; Sheridan & McLaughlin, 2014; Teicher et al., 2012). This therefore suggests a pathway by which early threat exposure reduces hippocampal and prefrontal volume and function in favour of greater activity in the amygdala, creating a system by which an individual is more sensitive and biased towards future threat exposure. Furthermore, the Dimensional Model of Adversity illustrates the pathways by which socioeconomic factors impair cognitive function. Greater deprivation is predictive of reduced synaptic pruning, which impairs the ability to form social and cognitive associations in development and causes cortical thinning in the lateral and medial prefrontal cortices. Threat exposure is predictive of reduced hippocampal and prefrontal volume as well as increased amygdala activity, resulting in a system by which cognitive resources are biased and diverted towards new potential threats.

### Pressing issues and future directions

I believe *the Bucket Model of Socioeconomic Working Memory Disparities* proposes an important new avenue to view the impact of socioeconomic status on cognition. At the same time, there are a number of issues that require clarification. First, and perhaps most important, is to integrate *the Bucket Model of Socioeconomic Working Memory Disparities* and *the Dimensional Model of Adversity*. The developmental influences of threat and deprivation are quite clear, exerting long‐lasting influences on the structure and function of the prefrontal cortex (McLaughlin et al., 2014, 2019; Sheridan et al., 2022; Sheridan & McLaughlin, 2014). Rather than disputing this view, the proposed model instead suggests an additional method by which to examine the effects of inequality. *The Dimensional Model of Adversity* focuses almost entirely on adverse childhood experiences through the presence or absence of environmental factors. Although childhood socioeconomic status is highly predictive of socioeconomic status later in life (Duncan et al., 2017), one can experience financial adversity in adulthood without having experienced it in childhood and vice versa. Furthermore, as previously discussed, internal factors such as worry, social comparison and rumination are also strongly predictive of cognitive outcomes (Connolly et al., 2014; Hayes et al., 2008; Kraus, 2018; Levens et al., 2009; Sari et al., 2017; Smith, 2013; van de Ven, 2017; L. Zhang et al., 2019, 2021). *The Bucket Model of Socioeconomic Working Memory Disparities* therefore provides a novel lens to examine internal social/affective factors, whereas *the Dimensional Model of Adversity* provides a means to examine external environmental factors. Within populations where individuals experience both internal and external facets of financial adversity, both models can be used in conjunction with one another to examine distinct but related pathways by which neural development contributes to increased threat sensitivity, which in turn may increase one's susceptibility to rumination and worry (Figure 3). This also links work examining childhood socioeconomic status and adult socioeconomic status, as childhood levels of deprivation and threat might influence neural development, which influences one's susceptibility to worry and rumination on socioeconomic‐related worries in adulthood via changes to threat sensitivity.

**FIGURE 3:**
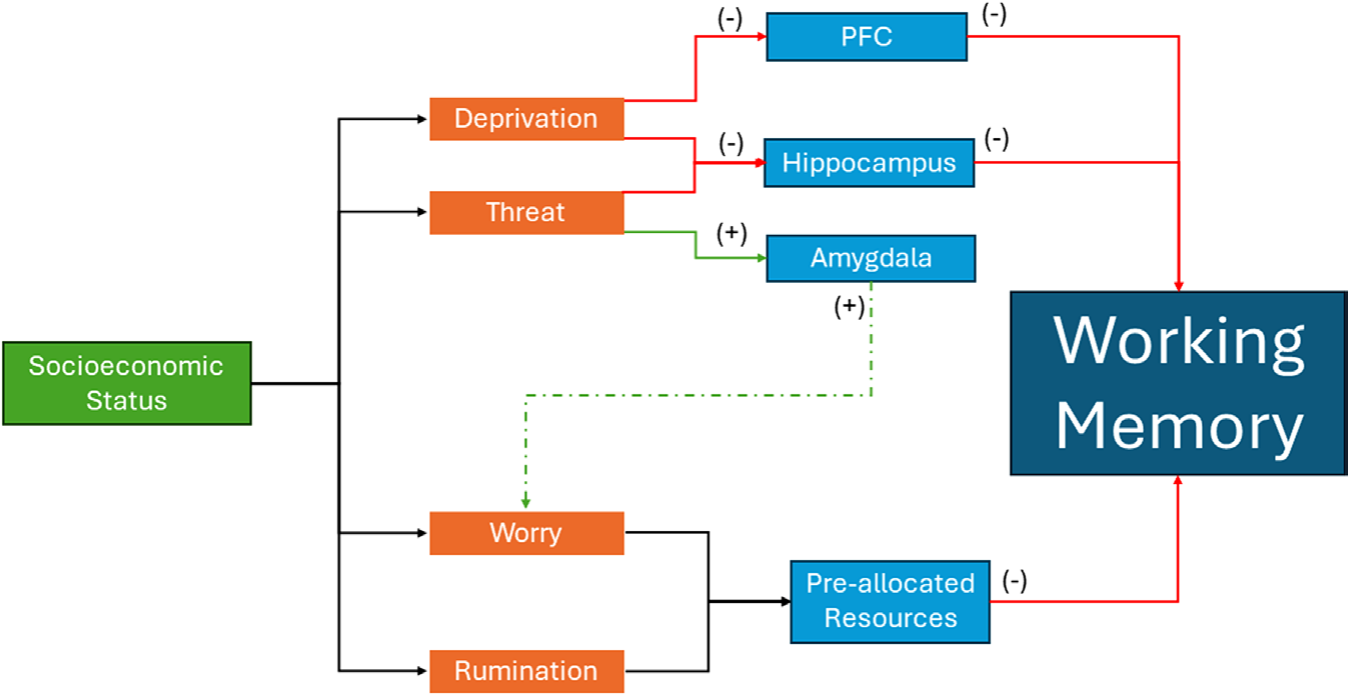
Integrated model of disparity and working memory. A proposed attempt to integrate the proposed model and the dimensional model of adversity.

A second important future direction is whether distinct patterns of adversity differentially contribute to worry, which in turn impairs working memory. Childhood threat exposure is associated with greater basal amygdala activity along with increased sensitivity to future threat exposure (Raineki et al., 2012; Sheridan & McLaughlin, 2014). This would potentially suggest that childhood threat exposure would be predictive of greater susceptibility to worry when faced with similar financial threats later in adulthood, whereas prior deprivation might not be. Additionally, amygdala activity may be linked to working memory performance. However, the exact nature of that link is somewhat nuanced. Amygdala activity has been found to be down‐regulated during low‐load working memory tasks but is associated with better working memory performance in high‐load tasks (de Voogd & Hermans, 2022; Li et al., 2023; Schaefer et al., 2006). Furthermore, additional work has suggested that a mismatch between adversity experienced during childhood and adversity experienced during adulthood is more predictive of dysfunction than allostatic load (Nederhof & Schmidt, 2012). Future research should therefore examine whether similar shifts in resource allocation in working memory tasks are observed in people who experience a mismatch in childhood/adulthood financial adversity as in those who experience a consistent level of adversity throughout their life.

Third, although some discussion has been given to broader cognitive processes, the proposed model focuses primarily on working memory processes. However, cognition is not monolithic and the proposed working memory mechanisms are likely to be influenced by changes in other cognitive processes. For example, there is evidence that financial scarcity elicits greater focus on some problems and attentional neglect of others (Shah et al., 2012). Within the proposed model, this would result in prioritized problems occupying working memory resources and task‐relevant processes being less prioritized. This difference in attentional capture, however, may be better explained by differences in attentional processes or differences in cognitive control. Similarly, work has found that with low‐SES participants, financially relevant worries arise more frequently and require greater effort to suppress (Shah et al., 2018), aligning with the proposed explanation for working memory deficits but differing in their point of origin and intervention. Finally, there is evidence that these influences may be rooted in decision‐making processes. Work by Chang et al. (2022) suggests that participants experiencing neighbourhood‐level scarcity may view finance‐related cues as more salient, prioritizing them more heavily when making decisions. This is similar to work examining scarcity‐related shifts in decision‐making (Sheehy‐Skeffington, 2020), and would then suggest a heavy role of decision‐making priorities in influencing items that are maintained in working memory. Considered together, these findings provide evidence for the proposed mechanism: financially relevant concerns are given preferential cognitive resources, resulting in relative deficits in working memory performance as socioeconomic worries occupy working memory resources. However, these findings also raise questions about potential points of intervention. The Bucket Model focuses on working memory, and suggests that reducing socioeconomic worry might free up cognitive resources. Work concerning inhibitory control would suggest that intervention should focus on keeping socioeconomic worries out of mind when attending to them is not beneficial, resulting in working memory resources not being occupied in the first place; whereas work examining attentional capture and decision‐making might look at influencing the relevant salience of task‐relevant and financially relevant information. Disentangling the roles of attention, decision‐making, inhibitory control and working memory from one another is critical in gaining a greater understanding of cognitive processes within disadvantaged individuals, and is vital for examining and developing effective points of intervention.

Finally, socioeconomic factors are heavily entrenched in culture. Individuals with similar objective socioeconomic status (i.e., income, access to resources, education) may experience completely different levels of worry, rumination and social comparison. Therefore, future research should examine whether links between socioeconomic status, socioeconomic worry and working memory resources are similar in Western, educated, industrialized, rich and democratic (WEIRD) populations vs. non‐WEIRD populations. Although some evidence for this association comes from Eastern cultures (Chan et al., 2018; Huang et al., 2016; K. Lee, 2023; L. Zhang et al., 2019, 2021), direct cultural comparison could illuminate whether the influences of socioeconomic worry are a generalizable mechanism or a distinct product of Western society. Furthermore, typical working memory tasks may vary in how culturally appropriate they are, both across nationalities and across socioeconomic dimensions (Alper et al., 2025; Silverman et al., 2023). In addition to examining the role of socioeconomically‐relevant stimuli, future work should also seek to examine whether typical working memory tasks are biased towards higher socioeconomic‐status individuals in order to disentangle the role of task relevance from task bias.

### Testable predictions

A number of testable predictions can be derived from this model. First, the proposed model suggests that socioeconomic worries occupy working memory resources because low socioeconomic status individuals can less easily shift from them to task‐relevant processes and stimuli. Should this be the case, experimentally induced socioeconomic worry should exert a greater influence on working memory processes than induced general worry within low socioeconomic participants, as relevant information from the induced socioeconomic worry would be given preferential attention over task‐relevant stimuli. Future research should therefore seek to use writing‐based affect inductions to preferentially examine the effects of socioeconomic worry and general worry within a socioeconomically diverse sample.

Similarly, should socioeconomic worry be the driving factor behind working memory deficits, interventions focused on reducing worry should yield improvements in working memory performance. Mindfulness‐based interventions such as mediation have been found to reduce worry (Course‐Choi et al., 2017; Delgado et al., 2010; Jain et al., 2007; Wolkin, 2015). Furthermore, mindfulness‐based mediation has also been found to improve working memory capacity in adolescents (Quach et al., 2016; van Vugt & Jha, 2011). Similarly, cannabidiol (CBD) has emerged as a potential treatment for anxiety and worry, and has been found to have potential benefits on cognitive functioning in multiple samples (Wieghorst et al., 2022; Yndart Arias et al., 2024). If working memory disparities between low and high socioeconomic status individuals are indeed due to the effects of worry, we would expect changes in worry to mediate the link between interventions and working memory improvements. Furthermore, we would expect these changes to be more beneficial for low socioeconomic status individuals, as they experience greater baseline worry.

Finally, if observed differences in working memory are due to a difference in resource allocation towards socioeconomic worries, no such difference should be observed for tasks relevant to socioeconomic worries. We would observe a task‐relevance effect such that financial cues would divert resources away from the task when the task information is irrelevant to financial concerns, but no such effect on a task where task information is relevant to financial concerns. Furthermore, we would expect differences in task‐relevance to be moderated by inhibitory control, as inhibition would influence how effectively a participant could allocate resources away from task‐irrelevant information.

## SUMMARY AND CONCLUSION

As described above, low socioeconomic status individuals face a plethora of objective and subjective barriers to success that contribute to a cycle of perpetual inequality. Cognitive deficits, especially impairments in working memory, create a uniquely difficult barrier. Working memory is critical for developing useful life skills, reading comprehension, language generation, planning and decision‐making. It is therefore vital to understand the mechanisms by which these impairments arise in order to reduce socioeconomic inequality.

The existing literature largely viewed these deficits through developmental pathways. Environmental threats and increased deprivation exert distinct influences on neural development, hampering the development of associations key to proper working memory later and life and resulting in increased sensitivity to threat. To elucidate the impact of socioeconomic status on working memory, I reviewed evidence that socioeconomic worry and social comparison influence allocation of working memory resources. Based on this work, I proposed the *Bucket Model of Socioeconomic Working Memory Disparities*, which posits that socioeconomic differences in working memory performance are due the preferential allocation of working memory resources towards socioeconomic worry (i.e., “a bucket that is half full”) rather than a reduction in cognitive resources (i.e., “a smaller bucket”).

In conclusion, the research reviewed here provides an important theoretical framework to view concurrent influences of socioeconomic status on working memory, offering an integrative model that can exist in conjunction with existing developmental frameworks.

## AUTHOR CONTRIBUTIONS

**Colton L. Hunter:** Conceptualization; writing – original draft; validation; visualization; writing – review and editing; supervision; project administration.

## CONFLICT OF INTEREST STATEMENT

The author reports no conflicts of interest.

## Data Availability

Data sharing not applicable to this article as no datasets were generated or analysed during the current study.
